# Obstruction in the third portion of the duodenum due to a diospyrobezoar: a case report

**DOI:** 10.1186/s12893-017-0308-9

**Published:** 2017-11-29

**Authors:** Yukinori Yamagata, Kazuyuki Saito, Kosuke Hirano, Yawara Kubota, Ryuji Yoshioka, Takashi Okuyama, Emiko Takeshita, Nobumi Tagaya, Shinichi Sameshima, Tamaki Noie, Masatoshi Oya

**Affiliations:** Department of Surgery, Dokkyo Medical University Koshigaya Hospital, 2-1-50 Minami-Koshigaya, Koshigaya City, Saitama, 343-8555 Japan

**Keywords:** Duodenal obstruction, Diospyrobezoar, Gastrectomy

## Abstract

**Background:**

Duodenal obstruction occurs mainly due to physical lesions such as duodenal ulcers or tumors. Obstruction due to bezoars is rare. We describe an extremely rare case of obstruction in the third portion of the duodenum caused by a diospyrobezoar 15 months after laparoscopic distal gastrectomy for early gastric cancer.

**Case presentation:**

A 73-year-old man who underwent laparoscopic distal gastrectomy for early gastric cancer 15 months before admission experienced abdominal distension and occasional vomiting. The symptoms worsened and ingestion became difficult; therefore, he was admitted to our department. Computed tomography (CT) performed on admission revealed a solid mass in the third portion of the duodenum and dilatation of the oral side of the duodenum and remnant stomach. Esophagogastroduodenoscopy (EGD) revealed a bezoar deep in the third portion of the duodenum. We could neither remove nor crush the bezoar. At midnight on the day of EGD, he experienced sudden abdominal pain. Repeat CT revealed that the bezoar had vanished from the duodenum and was observed in the ileum. Moreover, small bowel dilatation was observed on the oral side of the bezoar. Although CT showed neither free air nor ascites, laboratory data showed the increase of leukocyte (8400/μL) and C-reactive protein (18.1 mg/dL), and abdominal pain was severe. Emergency surgery was performed because conservative treatment was considered ineffective. We tried advancing the bezoar into the colon, but the ileum was too narrow; therefore, we incised the ileum and removed the bezoar. The bezoar was ocher, elastic, and hard, and its cross-section was uniform and orange. The postsurgical interview revealed that the patient loved eating Japanese persimmons (*Diospyros kaki*); therefore, he was diagnosed with a diospyrobezoar. His postoperative progress was good and without complications. He left the hospital 10 days after surgery. EGD performed 4 weeks after surgery revealed no abnormal duodenal findings.

**Conclusions:**

We describe a rare case of obstruction in the third portion of the duodenum caused by a diospyrobezoar 15 months after laparoscopic distal gastrectomy with Billroth I reconstruction for early gastric cancer.

## Background

Duodenal obstruction occurs mainly due to physical lesions such as duodenal ulcers or tumors [1]. Reports documented duodenal obstruction due to bezoars are relatively rare [2–11]. Furthermore, obstruction in the third portion of the duodenum due to a bezoar is extremely rare [2, 12, 13]. We describe a rare case of obstruction in the third portion of the duodenum caused by a diospyrobezoar 15 months after laparoscopic distal gastrectomy with Billroth I reconstruction for early gastric cancer.

## Case presentation

A 73-year-old man who had undergone laparoscopic distal gastrectomy with Billroth I reconstruction for early gastric cancer 15 months before experiencing abdominal distension and occasional vomiting was admitted to our hospital. The patient had undergone follow-up computed tomography (CT) and esophagogastroduodenoscopy (EGD) for 1 year after surgery (2 months before admission), during which time no abnormal findings, including the previously observed dilatation of the bile and pancreatic ducts, were found.

**Fig. 1:**
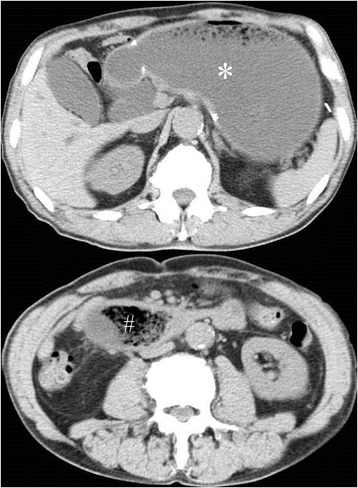
Computed tomography (CT) on admission. CT revealed dilatation of the remnant stomach (*) and a bezoar in the third portion of the duodenum (#)

**Fig. 2:**
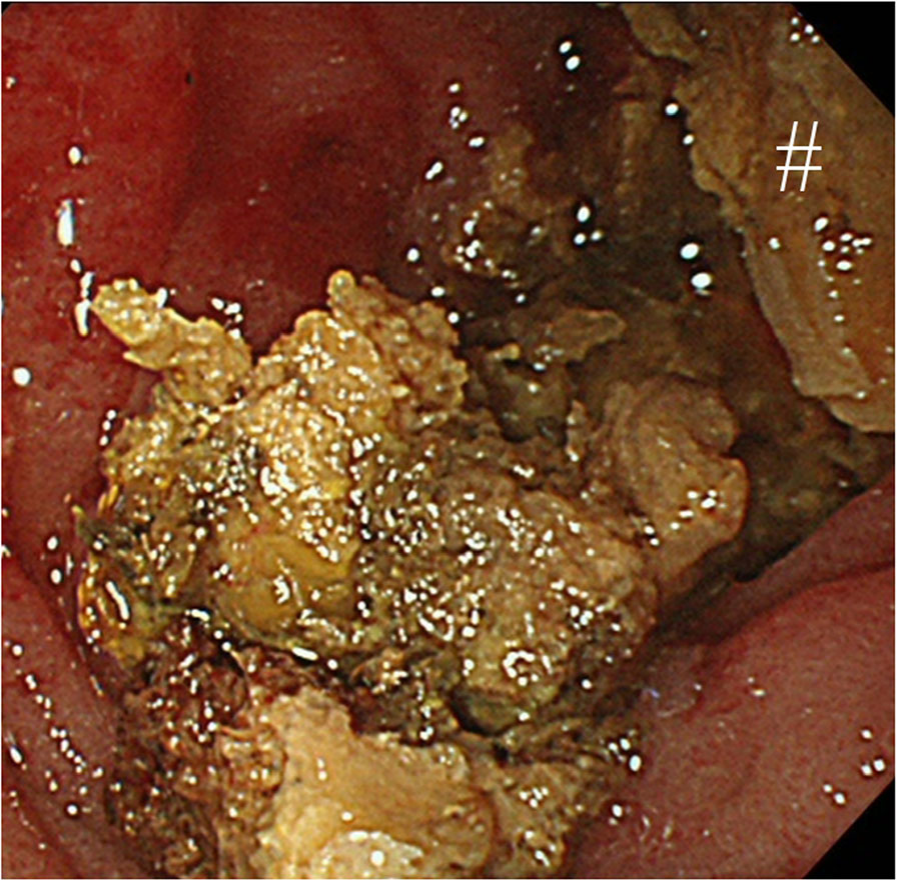
Esophagogastroduodenoscopy (EGD) on admission. EGD revealed a bezoar deep in the third portion of the duodenum (#)

**Fig. 3:**
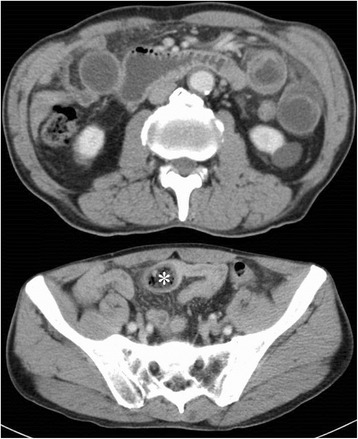
Computed tomography performed the day after esophagogastroduodenoscopy. The bezoar had disappeared from the duodenum and moved through the ileum (*)

**Fig. 4:**
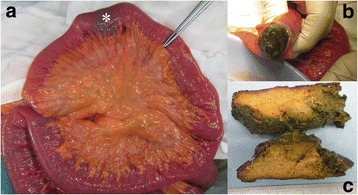
Intraoperative findings and bezoar. **a** The bezoar (*) was observed in the ileum. **b** The ileum was incised and the bezoar was removed. **c** The bezoar was ocher, elastic, and hard, and its cross-section was uniform and orange

Symptoms worsened and ingestion became difficult. On admission, blood tests showed a slightly inflammatory reaction, and CT revealed a solid mass in the third portion of the duodenum and dilatation of the oral side of the duodenum and remnant stomach (Fig. 1). EGD revealed a bezoar deep in the third portion of the duodenum (Fig. 2). The bezoar collapsed while trying to grasp it with forceps. We then tried crushing the bezoar with the forceps, but it was too deep to crush completely. We were also unable to search the duodenum on the other side of the bezoar. At midnight on the day of the EGD, he experienced sudden abdominal pain. Blood tests performed the next morning showed a significant increase in inflammatory response. Therefore, CT was repeated. The bezoar had disappeared from the duodenum but was observed in the ileum (Figs. 3 and 4a). Moreover, small bowel dilatation was observed on the oral side of the bezoar. Although CT showed no free air or ascites, laboratory data showed the increase of leukocyte (8400/μL) and C-reactive protein (18.1 mg/dL), and abdominal pain was severe. Emergency surgery was performed because conservative treatment was considered ineffective. Although the small intestine was expanded and edematous on the oral side of the bezoar, no perforation or necrosis was observed. We tried advancing the bezoar into the colon, but the ileum was too narrow; therefore, we incised the ileum and removed the bezoar (Fig. 4b). The bezoar was ocher, elastic, and hard, and its cross-section was uniform and orange (Fig. 4c). We found no abnormal duodenal findings on palpation. The postsurgical interview revealed that there was the tree of persimmon in the garden of his house and he loved eating Japanese persimmons (*Diospyros kaki*) and seemed to eat 30-40 persimmons in one season; therefore, a diospyrobezoar was diagnosed. His postoperative progress was good and without complications. He left the hospital 10 days after surgery. EGD performed 4 weeks after surgery revealed no abnormal duodenal findings.

## Discussion and conclusions

Bezoars are solid masses of ingested foreign materials found in the lumen of the gastrointestinal tract and are named according to their composition, such as phytobezoars (food fibers), trichobezoars (hair), and pharmacobezoars (drugs) [14]. The diospyrobezoar is a type of phytobezoar caused by ingestion of persimmons (*Diospyros kaki*) [14]. Persimmons are rich in fiber and *shibuol*, which is a type of tannin [14]. When *shibuol* comes in contact with gastric juice, polymerization of the persimmon fiber occurs and contributes to the formation of diospyrobezoars [14].

Moreover, gastrectomy is associated with the formation of diospyrobezoars [14]. Gastrectomy may reduce the motion of the remnant stomach and delay gastric emptying, leading to easy formation of bezoars [14]. Our patient had a history of gastrectomy and loved to eat persimmons, and it was suggested that the diospyrobezoar formed in the remnant stomach or duodenum.

We searched for articles with ‘duodenal obstruction’ and ‘bezoar’ as keywords in PubMed, 93 articles were hit. Filtered 93 articles by the publication date (in this decade) and the language (English), 17 articles remained. In addition, excluded those of animals, duodenal obstruction due to trauma, other than duodenal obstruction and reviews, 10 articles remained [2–11]. In these 10 articles, there were 11 cases of duodenal obstruction caused by bezoar. Among these 11 cases, 2 cases were so-called Rapunzel Syndrome (caused by trichobezoar) [3, 4], 1 case was bezoar resulting from industrial material [8], and other 9 cases were phytobezoar [2, 5–7, 9–11]. The location of bezoars were as follows: 6 for first portion [3, 6, 7, 9–11], 2 for second portion [7, 8], 1 for third portion [2], 1 for diverticulum of the duodenum [5], and 1 for entire duodenum [4].

In our case, bezoar was located in the third portion of the duopdenum. Leanness due to gastrectomy causes superior mesenteric artery (SMA) syndrome, which involves compression of the third portion of the duodenum between the aorta and the SMA. The diospyrobezoar formed in the remnant stomach or in the duodenum, suggesting that it was caught in the third portion of the duodenum and suggesting SMA syndrome. Among previous 11 cases, only a case was similar to our case [2]. We searched furthermore articles for ‘SMA syndrome’ and ‘bezoar’, we could find only 2 articles [12, 13]. This case suggested to be rare.

As a treatment for trichobezoar, surgical methods, endoscopic methods (crushing, removal) and dissolution therapy by Coca-Cola are known. Dissolution therapy by Coca-Cola was introduced by Ladas et al. in 2002 [15]. Recent systematic review have shown that Coca-Cola therapy (alone or combined with endoscopic therapy) for trichobezoar was effective in 91.3% of the cases [16]. In our case, initially, the bezoar existed in the third portion of the duodenum, Coca-cola lavage therapy was considered to be difficult to implement. After the bezoar fell in the ileum, the patient had severe abdominal pain, and the conservative treatment considered ineffective. Hence we selected surgical treatment.

We describe an extremely rare case of obstruction in the third portion of the duodenum caused by a diospyrobezoar 15 months after laparoscopic distal gastrectomy with Billroth I reconstruction for early gastric cancer.

## Abbreviations

CT: Computed tomography
D: Esophagogastroduodenoscopy
SMA: Superior mesenteric artery

## References

[1] Carbo AI, Sangster GP, Caraway J, Heldmann MG, Thomas J, Takalkar A (2014). Acquired constricting and restricting lesions of the descending duodenum. Radiographics.

[2] Fan S, Wang J, Li Y (2016). An unusual cause of duodenal obstruction: persimmon Phytobezoar. Indian J Surg.

[3] Caiazzo P, Di Lascio P, Crocoli A, Del Prete I (2016). The Rapunzel syndrome. Report of a case. G Chir.

[4] Koushk Jalali B, Bingöl A, Reyad A (2016). Laparoscopic Management of Acute Pancreatitis Secondary to Rapunzel syndrome. Case Rep Surg.

[5] Kim JH, Chang JH, Nam SM, Lee MJ, Maeng IH, Park JY (2012). Duodenal obstruction following acute pancreatitis caused by a large duodenal diverticular bezoar. World J Gastroenterol.

[6] Guner A, Kahraman I, Aktas A, Kece C, Reis E (2012). Gastric outlet obstruction due to duodenal bezoar: a case report. Int J Surg Case Rep.

[7] Chao HC, Chang KW, Wang CJ (2012). Intestinal obstruction caused by potato bezoar in infancy: a report of three cases. Pediatr Neonatol.

[8] Selcuk H, Unal H, Korkmaz M, Yilmaz U (2009). Subacutely formed bezoar resulting from accidentally ingested industrial material. J Chin Med Assoc.

[9] Hussain A, Mahmood H, Singhal T, El-Hasani S (2008). An unusual cause of gastric outlet obstruction during percutaneous endogastric feeding: a case report. J Med Case Rep.

[10] Singh SK, Marupaka SK (2007). Duodenal date seed bezoar: a very unusual cause of partial gastric outlet obstruction. Australas Radiol.

[11] Chiu HH, Li JH (2007). Gastric outlet obstruction caused by a dumbbell-shaped phytobezoar impacted in a deformed duodenal bulb. Gastrointest Endosc.

[12] Doski JJ, Priebe CJ, Smith T, Chumas JC (1995). Duodenal trichobezoar caused by compression of the superior mesenteric artery. J Pediatr Surg.

[13] Fuhrman MA, Felig DM, Tanchel ME (2003). Superior mesenteric artery syndrome with obstructing duodenal bezoar. Gastrointest Endosc.

[14] de Toledo AP, Rodrigues FH, Rodrigues MR, Sato DT, Nonose R, Nascimento EF (2012). Diospyrobezoar as a cause of small bowel obstruction. Case Rep Gastroenterol.

[15] Ladas SD, Triantafyllou K, Tzathas C, Tassios P, Rokkas T, Raptis SA (2002). Gastric phytobezoars may be treated by nasogastric Coca-Cola lavage. Eur J Gastroenterol Hepatol.

[16] Ladas SD, Kamberoglou D, Karamanolis G, Vlachogiannakos J, Zouboulis-Vafiadis I (2013). Systematic review: Coca-Cola can effectively dissolve gastric phytobezoars as a first-line treatment. Aliment Pharmacol Ther.

